# Changes in cognitive function, synaptic structure and protein expression after long-term exposure to 2.856 and 9.375 GHz microwaves

**DOI:** 10.1186/s12964-022-01011-1

**Published:** 2023-02-13

**Authors:** Hui Wang, Yu Liu, Yunbo Sun, Ji Dong, Xinping Xu, Haoyu Wang, Xuelong Zhao, Jing Zhang, Binwei Yao, Li Zhao, Shuchen Liu, Ruiyun Peng

**Affiliations:** Beijing Institute of Radiation Medicine, Beijing, 100850 China

**Keywords:** Microwave, hippocampus, Learning and memory function, Exosomes, Proteomics

## Abstract

Health hazards from long-term exposure to microwaves, especially the potential for changes in cognitive function, are attracting increasing attention. The purpose of this study was to explore changes in spatial learning and memory and synaptic structure and to identify differentially expressed proteins in hippocampal and serum exosomes after long-term exposure to 2.856 and 9.375 GHz microwaves. The spatial reference learning and memory abilities and the structure of the DG area were impaired after long-term exposure to 2.856 and 9.375 GHz microwaves. We also found a decrease in SNARE-associated protein Snapin and an increase in charged multivesicular body protein 3 in the hippocampus, indicating that synaptic vesicle recycling was inhibited and consistent with the large increase in presynaptic vesicles. Moreover, we investigated changes in serum exosomes after 2.856 and 9.375 GHz microwave exposure. The results showed that long-term 2.856 GHz microwave exposure could induce a decrease in calcineurin subunit B type 1 and cytochrome b-245 heavy chain in serum exosomes. While the 9.375 GHz long-term microwave exposure induced a decrease in proteins (synaptophysin-like 1, ankyrin repeat and rabankyrin-5, protein phosphatase 3 catalytic subunit alpha and sodium-dependent phosphate transporter 1) in serum exosomes. In summary, long-term microwave exposure could lead to different degrees of spatial learning and memory impairment, EEG disturbance, structural damage to the hippocampus, and differential expression of hippocampal tissue and serum exosomes.

**Supplementary Information:**

The online version contains supplementary material available at 10.1186/s12964-022-01011-1.

## Introduction

With the development of science and technology, an increasing number of different microwave emission sources have become common in our daily lives. As a result, increasing attention has been given to the health hazards caused by microwaves [1–3]. Among them, S-band microwaves, and X-band microwaves are widely used in aircraft, ship and ground communication or meteorological monitoring. For example, most aircraft and ships are equipped with X-band radar systems because these systems often use smaller antennas and can produce smaller wavelengths than other radar systems, which makes them especially useful for communication and short-distance weather observation. In addition, the waves generated by S-band radar systems are not easily attenuated; this property increases their penetration when they are used for close and long-range weather observation or communication. People who engaged in related work or live near the above radar systems might be exposed to their radiation output for long periods. Existing studies have shown that microwave radiation could lead to central nervous system (CNS) injuries in animals, especially spatial learning and memory function [4, 5]. Therefore, we believe it is necessary to study the damage caused by long-term exposure to microwaves with different frequencies.

The hippocampus is closely related to learning and memory function and is involved in different types of memory [6]. Meanwhile, previous studies indicated that the hippocampus was considered a sensitive brain region for microwave radiation [7–9]. Synaptic plasticity is one of the cellular mechanisms of learning and memory [10–12]. The structural plasticity of synapses includes the formation and elimination of synapses, as well as structural changes in the strength of synaptic connections [13, 14], which can directly affect information exchange among cells.

Proteins, as molecules that carry out biological activities, can be analyzed by high-throughput proteomic analysis to provide potentially sensitive molecules for understanding the mechanism. However, current studies on microwave-induced injuries have not provided the specific signaling pathways or underlying mechanisms [15–17]. Proteomics provides a way to quantify a large number of proteins after high-throughput sequencing [18]. In addition to finding differentially expressed proteins in certain brain areas, such as the hippocampus, intercellular communication also plays an important role in learning and memory functions [19, 20].

Exosomes are extracellular membrane vesicles (EMVs) that can transmit information between cells via functional biomolecules, such as proteins, mRNAs, microRNAs, noncoding RNAs, DNA and lipids. Exosomes can be released by many cells in the nervous system and cross the blood‒brain barrier to enter the peripheral blood. Studies have shown that functional biomolecules in exosomes are involved in the development and pathological processes of the nervous system, such as amyloid precursor protein, brain-derived neurotrophic factor for neuronal development, and cystatin C protein in Alzheimer’s disease [21–23]. In this study, we developed a model of long-term microwave exposure at different frequencies to assess alterations in spatial learning and memory and hippocampal structure. Then, we screened and quantified sensitive proteins in the hippocampus and serum exosomes by proteomic assays and the pattern recognition method (PRM).

Through this study, we wished to clarify the damage characteristics and sensitive molecules after long-term microwave exposure to develop future therapeutic measures.

## Materials and methods

### Animals and microwave exposure

A total of 105 male Wistar rats (200 ± 20 g, 6–8 weeks) were purchased from Beijing Vital River Laboratory Animal Technology Co., Ltd. (Beijing, China) and maintained in a specific pathogen-free (SPF) grade animal facility. All rats were maintained at 22 ± 2 ℃ and 55 ± 10% humidity on a 12/12 h light-dark cycle (lights on at 8 a.m.). Food and distilled water were freely available. All experiments were performed during 8 a.m. to 6 p.m. Throughout the experiment, two or three rats were kept in one cage. All protocols were approved by the Institutional Animal Care and Use Committee (IACUC-AMMS-2020-780).

To exclude the possible influence of body weight, the rats were divided according to their weight into 3 groups before microwave exposure: the sham group (group C); the 2.856 GHz microwave exposure group (group S); the 9.375 GHz microwave exposure group (group X). Rats in the two radiation groups received whole-body exposure with the 10 mW/cm^2^ microwaves of the S and X bands, respectively, for 6 weeks (6 min per day, 5 days per week). Group C was processed in the same manner as the exposure groups except for the microwave radiation (Additional file 1: Fig. 1).

### Body temperature monitoring

Five rats from each group were selected randomly and detected by the portable thermometer JM222 (Beijing ZhongWang Electronic Technology Co., Ltd. Beijing, China) before and immediately after microwave exposure to investigate the changes in core temperature.

### Electroencephalogram (EEG) recording

An MP-150 multichannel physiological recording and analysis system (BIOPAC Company, USA) was used to test the electroencephalogram at 6 h after the last microwave exposure.

Fifteen rats (n = 5 per group) were injected with 1% pentobarbital sodium (0.5 g/100 g, IP). When the rats were under mild anesthesia, the hair on the top of the head was removed and disinfected with 75% ethanol. The recording electrodes were fixed bilaterally on top of the scalp near the midline, and the reference electrodes were inserted in the right ear lobe. All electrodes were connected to the amplifier and recorded for 3 min in a quiet state. All rats underwent EEG recording in random order.

### Morris water maze (MWM) test

MWM is a behavioral experiment widely used to examine the spatial reference learning and memory abilities of rodents [24, 25]. In the experiment, a circular pool (150 cm in diameter) filled with water at 23 ± 2 ℃ was divided into four equal quadrants. The platform (12 cm in diameter and 15 cm in height) was submerged 1 cm below the water surface in the target quadrant. The swimming trials of all rats were digitally recorded by the Anymaze system (Stoelting Co., Illinois, USA). Thirty-six rats (n = 12 per group) were trained to find the submerged platform before microwave exposure for three consecutive days (a maximum duration of 60 s per trial). Regardless of whether the rat found the platform by itself or not within 60 s, it would stay on the platform for 15 s to remember the platform location. After microwave exposure, the navigation tests were performed at 6 h and 1–6 d. The average escape latency (AEL), defined as the average time that the rat took to reach the escape platform over four trials, was used to evaluate spatial learning and memory. To record the number of crossings, the probe trials were carried out at 7 d after microwave exposure.

### Observation of the hippocampal microstructure

At 6 h, 7 d, 14 d and 28 d after the last microwave exposure, 5 rats in each group were sacrificed by using 1% pentobarbital sodium solution for pathological examination. The brain of each rat was removed, and the left half of the brain was fixed in 10% formalin buffer solution. From each left half of the brain, tissue containing the hippocampus was embedded in paraffin and cut into 5 μm thick sections in the coronal plane. The sections were deparaffinized and rehydrated with different concentrations of xylene (Sinopharm Chemical Reagent Co., Ltd, China) and alcohol (Sinopharm Chemical Reagent Co., Ltd, China) and then dipped in hematoxylin (ZSGB-BIO, China) for 5 min. The sections were destained in 1% hydrohalic acid ethanol for 7 s and redyed in eosin (ZSGB-BIO, China) for 2 min. Following dehydration in an alcohol gradient, xylene clearance and cover slipping, stained brain sections were examined under a microscope, and hippocampal tissue was meticulously analyzed after whole-section scans were taken.

### Observation of the hippocampal ultrastructure

Sections of hippocampal tissue with a thickness of 1 mm from each group at 6 h after microwave exposure were dissected. After being fixed in 2.5% glutaraldehyde (Merck, Darmstadt, Germany), the specimens were sequentially processed with 1% osmium tetroxide (AppliChem, Gatersleben, Germany) graded ethyl alcohol and embedded in EPON618 (TAAB Laboratories Equipment, Berks, UK). The thin sections on copper meshes were stained with uranyl acetate and lead citrate (Advanced Technology & Industrial Co. Ltd., Hong Kong, China) for contrast. After being dried, the grids were viewed on a transmission electron microscope (TEM, H-7650, Tokyo, Japan).

The thickness of the postsynaptic densities was measured by the software package Reconstruct ([Version 1.1.0.0, 2007], available from the website https://synapseweb.clm.utexas.edu/software-0*).* In brief, after the scale ruler was corrected, the thickness of the postsynaptic densities of all synapses was measured.

### Dendritic spine density and maturation ratio analysis of neurons in the hippocampus

This experiment was carried out by the Golgi Fast Kit (#PK401, FD NeuroTechnologies, USA). In brief, the mixture of solutions A and B was prepared at a ratio of 1:1 and stored in the dark a day before sampling. Three rats in each group were sacrificed 6 h after the last microwave exposure, and the left whole brain was taken. After immersion in the mixture of A and B, the new mixture of A and B was replaced the next day. The tissue was immersed in the mixture of A and B for 4 weeks and then transferred to solution C and changed with solution C the next day. Slices of 100 μm were cut using a cryostat after the samples were stored in the dark for 7 days. Staining was performed according to the protocol in the instruction manual, and pictures were taken with a 100× optical microscope (Leica, Wetzlar, Germany). Finally, dendritic spine analysis of the distal dendrites of pyramidal cells and granule cells was performed by ImageJ software (v1.8.021, National Institutes of Health, USA).

### Quantitative proteomic analysis of hippocampal and serum exosomes

#### Total protein extraction of the hippocampus

At 6 h after the last microwave exposure, left hippocampus samples of all rats were separated and stored at − 80 °C. All samples (n = 5 per group) were cut individually and lysed in lysis buffer containing 50 mM NH_4_HCO_3_ (pH 7.4), 10 mM MgCl_2_, 7 M urea and 2 M thiourea, followed by 5 min of ultrasonication on ice. The lysate was centrifuged at 12,000×*g* for 15 min at 4 °C, and the supernatant was transferred to a clean tube.

#### Exosome isolation and identification

At 6 h after the last microwave exposure, serum of venous blood was collected and placed at − 80 °C (n = 5 per group). Then, exosomes were extracted by gradient centrifugation according to the following steps: (1) thaw the serum in a water bath at 25 °C and place it on ice; (2) centrifuge at 2000×*g* at 4 °C for 10 min and aspirate the supernatant; (3) centrifuge at 10,000×*g* at 4 °C for 30 min and keep the supernatant; (4) centrifuged at 110,000×*g* at 4 °C for 75 min and discard the supernatant; (5) resuspend the pellet with 1 ml PBS, dilute it further with PBS after resuspension, and filter it with a 0.22 μm membrane; (6) centrifuge at 110,000×*g* at 4 °C for 75 min and discard the supernatant; (7) resuspend the pellet in PBS and store it at − 80 °C.

Exosomes from each group were identified by Western blotting, nanoparticle tracking analysis (NTA), and morphology analysis by TEM. The detailed methods were as follows. (1) Isolated exosomes were tested for the positive exosomal markers CD9, CD63 and TSG101 (Abcam, UK) and the negative marker calnexin (Abcam, UK) by Western blot. (2) All samples were thawed in 25 °C water and diluted with PBS. The NTA detection was performed using a nanoparticle tracking analyzer (PARTICLE MATRIX, Germany) and ZetaView 8.04.02 software. (3) Five microliter exosome samples from the four groups were taken and dropped onto a copper net. After incubating for 5 min at room temperature, excess liquid was absorbed by absorbent paper. Then, 2% uranyl acetate was added to the copper net and incubated for 1 min. Excess liquid was absorbed. The samples were observed by TEM (Tecnai G2 Spirit BioTwin, FEI, USA).

#### Peptide preparation

Proteins in isolated hippocampus and exosome fractions were extracted, and the concentrations were measured by a BCA Protein Assay Kit (Pierce Biotechnology, USA) according to the manufacturer’s instructions. One hundred micrograms of protein from each sample was reduced with 10 mM DTT for 1 h at 56 °C and subsequently alkylated with sufficient iodoacetamide for 1 h at room temperature in the dark. The protein was digested with Trypsin Gold (Promega) at a 1:50 enzyme-to-substrate ratio. After 16 h of digestion at 37 °C, portions of the peptides from the samples were mixed equally. The mixture sample (mix-sample) and the remaining peptides (single-sample) were all desalted with a C18 cartridge to remove the high urea, and desalted samples were dried by vacuum centrifugation.

#### HPLC fractionation

The mixed samples were fractionated using a C18 column (Waters BEH C18 4.6 × 250 mm, 5 μm) on a Rigol L3000 HPLC operating at 1 mL/min, and the column oven was set as 50 °C. Mobile phases A (2% acetonitrile, adjusted pH to 10.0 using ammonium hydroxide) and B (98% acetonitrile, adjusted pH to 10.0 using ammonium hydroxide) were used to develop a gradient elution. The solvent gradient was set as follows: 5% B, 0 min; 8% B, 5 min; 18% B, 40 min; 32% B, 62 min; 95% B, 64 min; 95% B, 68 min; 5% B, 72 min. The eluates were monitored in the UV range (214 nm), collected into one tube per minute, and finally merged into 6 fractions. All fractions were dried under vacuum and reconstituted in 0.1% (v/v) formic acid (FA) in water. Add 0.2 µL of standard peptides to the fraction sample for subsequent analyses.

#### LC‒MS/MS analysis: DDA mode

For transition library construction, shotgun proteomics analyses were performed using a U3000 UHPLC system (Thermo Fisher, USA) coupled with an Orbitrap fusion mass spectrometer (Thermo Fisher, USA) in data-dependent acquisition (DDA) mode. A sample volume containing 1 µg of total peptides from the fraction sample reconstituted in 0.1% FA was injected onto a homemade C18 Nano-Trap column (2 cm×100 μm, 3 μm). Peptides were separated on an analytical column (25 cm×75 μm, 100 A) using a 120 min linear gradient from 0 to 100% eluent B (0.08% formic acid in 80% acetonitrile, 20% water) in eluent A (0.1% formic acid in water) at a flow rate of 600 nL/min. The detailed solvent gradient was as follows: 8% B, 0 min; 12% B, 7 min; 30% B, 55 min; 40% B, 65 min; 95% B, 66 min; 95% B, 80 min.

The Orbitrap Fusion mass spectrometer was operated in DDA mode using Xcalibur 3.0 software, and there was a single full-scan mass spectrum in the Orbitrap (300–1500 m/z, 120,000 resolution) followed by data-dependent MS/MS scans in an ion routing multipole at 30% normalized collision energy (HCD).

#### LC‒MS/MS analysis: DIA mode

The single sample was reconstituted in 0.1% formic acid, mixed with 0.2 µL standard peptides (iRT kit, Biognosys, Switzerland), and injected onto a U3000 UHPLC system (Thermo Fisher, USA) coupled with an Orbitrap Fusion Mass Spectrometer (Thermo Fisher, USA) operating in data-independent acquisition (DIA) mode. For DIA acquisition, the MS1 resolution was set to 60,000, and the MS2 resolution was set to 30,000. The m/z range covered 350 to 1350 m/z and varied over 42 cycles. The full-scan AGC target was set to 4 × 10^5^, and the injection time was 50 ms. The DIA settings were NCE 35%, target value 1 × 10^6^ and maximum injection time was set to auto to allow the mass spectrometer to always operate in the parallel ion filling and detection mode. The details of the 42 cycles can be found in Additional file 1: Table 1.

#### Data analysis

Data analysis and visualization of DDA and DIA data were performed using the Proteome Discoverer 2.4 (PD 2.4, Thermo Fisher, USA) platform, Biognosys Spectronaut version 13, and R statistical framework. DDA MS raw files were analyzed by PD software (version 2.4), and peak lists were searched against the protein database. The COG (Clusters of Orthologous Groups) and KEGG (Kyoto Encyclopedia of Genes and Genomes) databases were used to analyze the protein families and pathways. The differentially expressed proteins in the S and X groups compared to the C group samples were identified according to the chi-square test and one-way ANOVA, and the differentially expressed proteins between groups were screened and considered significant (fold change > 1.2, p value < 0.05). According to the results of the following three aspects of GO analysis, biological processes (BP), cellular components (CC), and molecular functions (MF), combined with KEGG pathways, we selected sets related to neuron structure, neuron function, synaptic plasticity, learning and memory for subsequent identification.

### Identification of differentially expressed proteins by the pattern recognition method (PRM)

The Orbitrap Fusion mass spectrometer was operated in the data-dependent acquisition mode using Xcalibur3.0 software, and there was a single full-scan mass spectrum in the Orbitrap (250–1450 m/z, 120,000 resolution) followed by the data-dependent MS/MS scans in an Ion Routing Multipole at 30% normalized collision energy (HCD). The MS data were processed using Skyline (v.3.6).

### Statistical analysis

Data are shown as the mean ± standard deviation. SPSS 25 was used to analyze all experimental data. A paired t test was used to analyze the effect of microwave radiation on the core temperature of rats. Two-way ANOVA with repeated measurements was performed to evaluate the results of the Morris water maze. All other results were analyzed by one-way ANOVA with a post hoc test when needed. In this study, Tukey’s honest significant difference (HSD) test was used for the post hoc test. The standard of significant differences was *p* < 0.05.

## Results

### Core temperature did not increase after microwave exposure

**Fig. 1:**
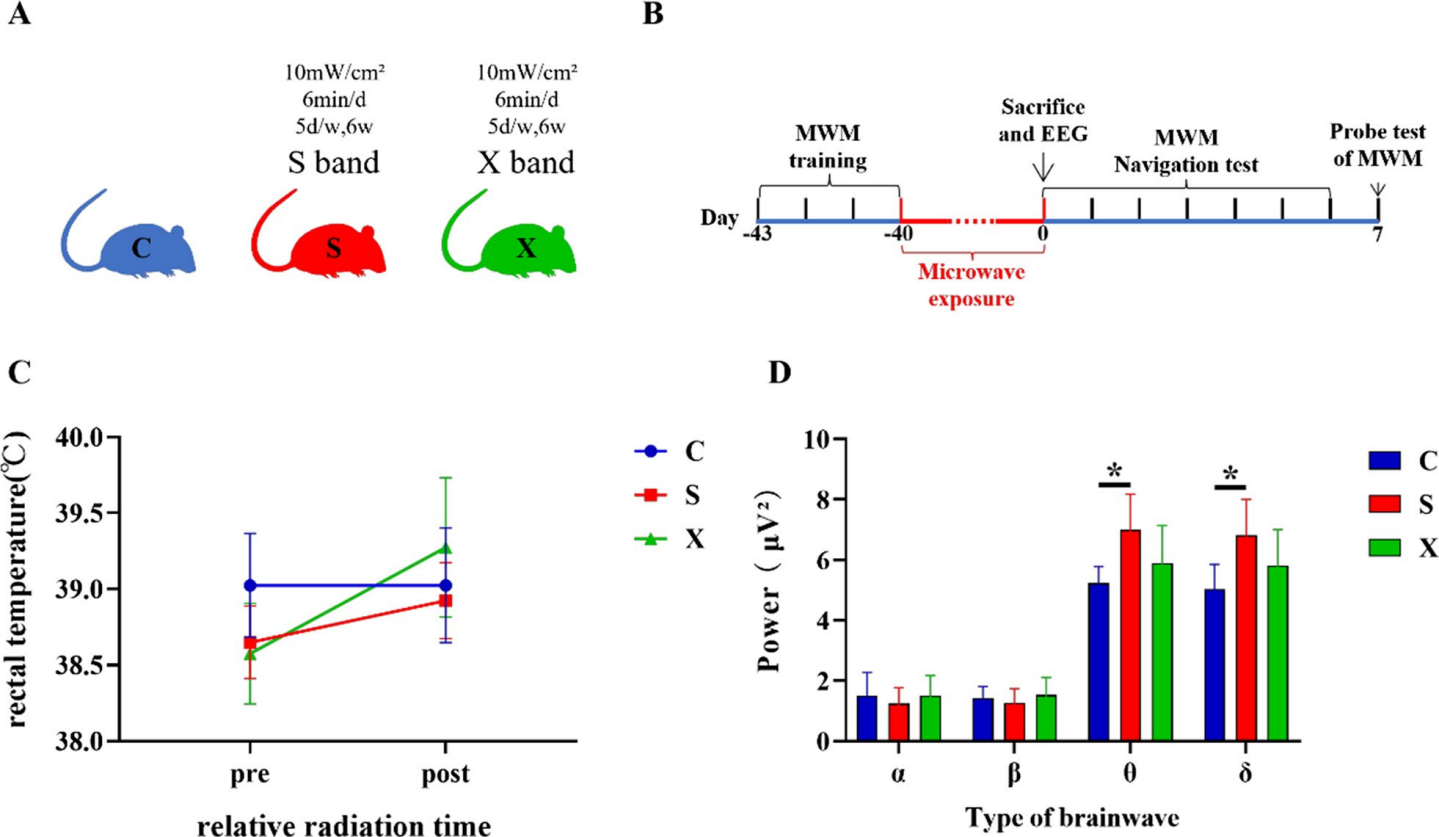
The rat model and changes in core temperature and EEG power after microwave exposure. **A** Schematic diagram of the rat model; **B** schematic diagram of the whole experiment; **C** change curve of rectal temperature before and after radiation; **D** α, β, θ and δ wave power at 6 h after microwave exposure; * indicates *p* < 0.05

The schematic diagrams of the different group treatments and the whole experimental process are shown in Fig. 1A, B. Since the entire exposure process lasted 6 weeks (6 min per time), we measured the core temperature of the rats before and immediately after each microwave radiation. There were no significant increases in rectal temperature (*p* = 0.1000, *p* = 0.128, *p* = 0.104) between the time points before and immediately after microwave exposure in any group, indicating that the effects of microwave radiation on the mice in this experiment were mainly nonthermal effects (Fig. 1C).

### EEG changes caused by microwave exposure

After one-way ANOVA, the power of θ and δ waves in the S group significantly increased compared with that in the C group at 6 h after the last exposure (*p* = 0.020, Fig. 1D; *p* = 0.023, Fig. 1D). At the same time, no significant differences were found among the groups in the power of α and β waves throughout the process.

The above results indicated that the excitability of the cortical EEG signals of the rats decreased in the S group.

### Spatial reference learning and memory abilities declined after microwave exposure

To determine the specific effects of long-term microwave exposure on spatial learning and memory function, Morris water maze tests were conducted.

**Fig. 2:**
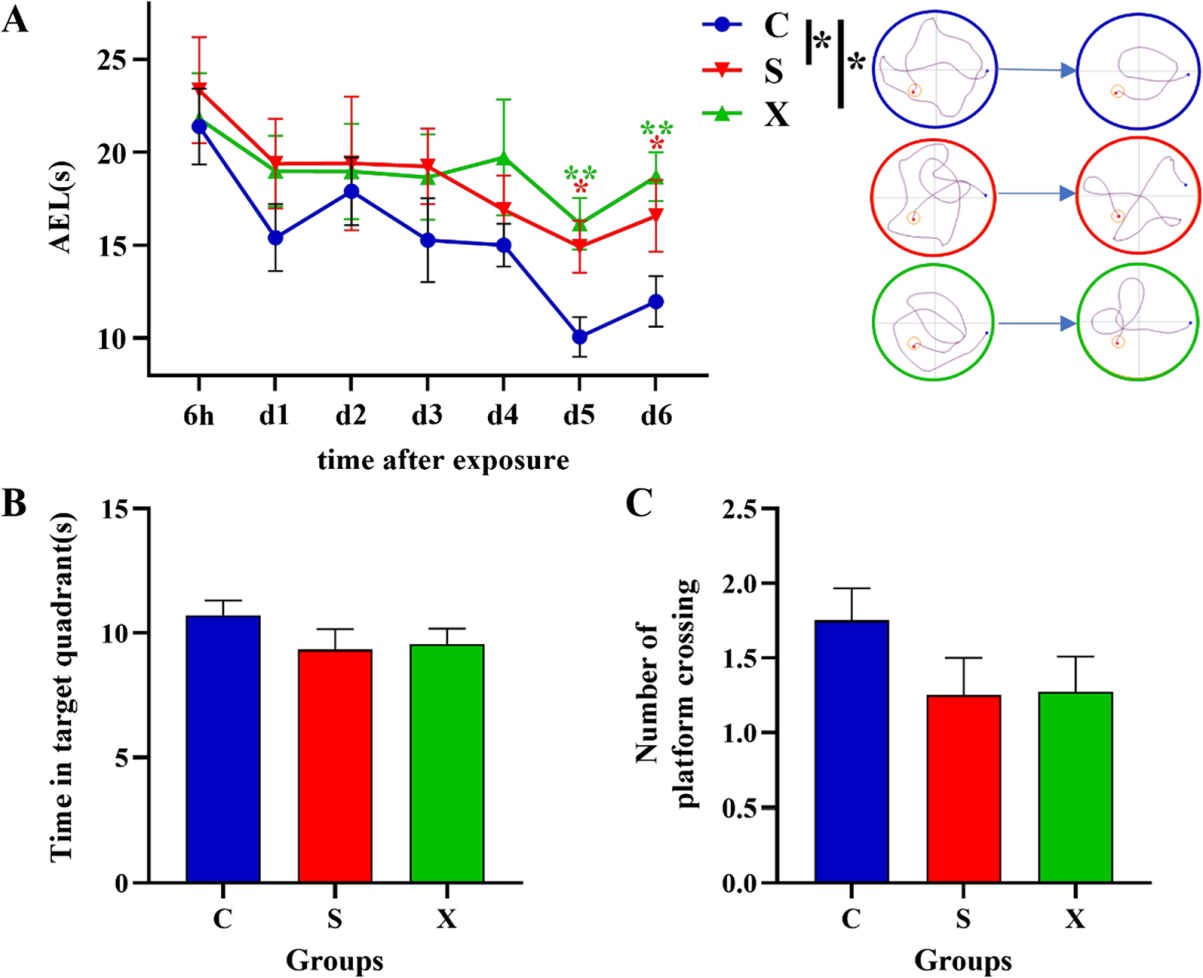
Changes in rat behavior after microwave exposure. **A** The learning curves of different groups of rats in the MWM after the last exposure. The right side was attached with a typical path of rats in each group at different time points. **B** Swimming time in the target quadrant for each group of rats in the probe test. **C** The number of platform crossings in the probe test for each group of rats; * indicates *p* < 0.05; ** indicates *p* < 0.01

In the MWM navigation tests, after two-way ANOVA with repeated measurements, in terms of the learning curve of different groups of rats, the learning speed of rats in both radiation groups was significantly lower than that in the C group (*p* = 0.035, *p* = 0.017). To further compare the daily performance, multiple comparisons were conducted. Compared with that of the C group, the AEL significantly increased in the S group at 5 d and 6 d after the last exposure (*p* = 0.012, *p* = 0.045, Fig. 2A), and the AEL significantly increased in the X group at 5 d and 6 d after the last exposure (*p* = 0.002, *p* = 0.005, Fig. 2A). By contrast, in the probe test, there were no significant differences among the groups of rats for the two commonly used metrics (Fig. 2B, C). The above results indicated that the long-term 2.856 and 9.375 GHz microwave radiation could damage the spatial reference learning and memory ability.

### Structural damage to the rat hippocampus after long-term microwave exposure

**Fig. 3:**
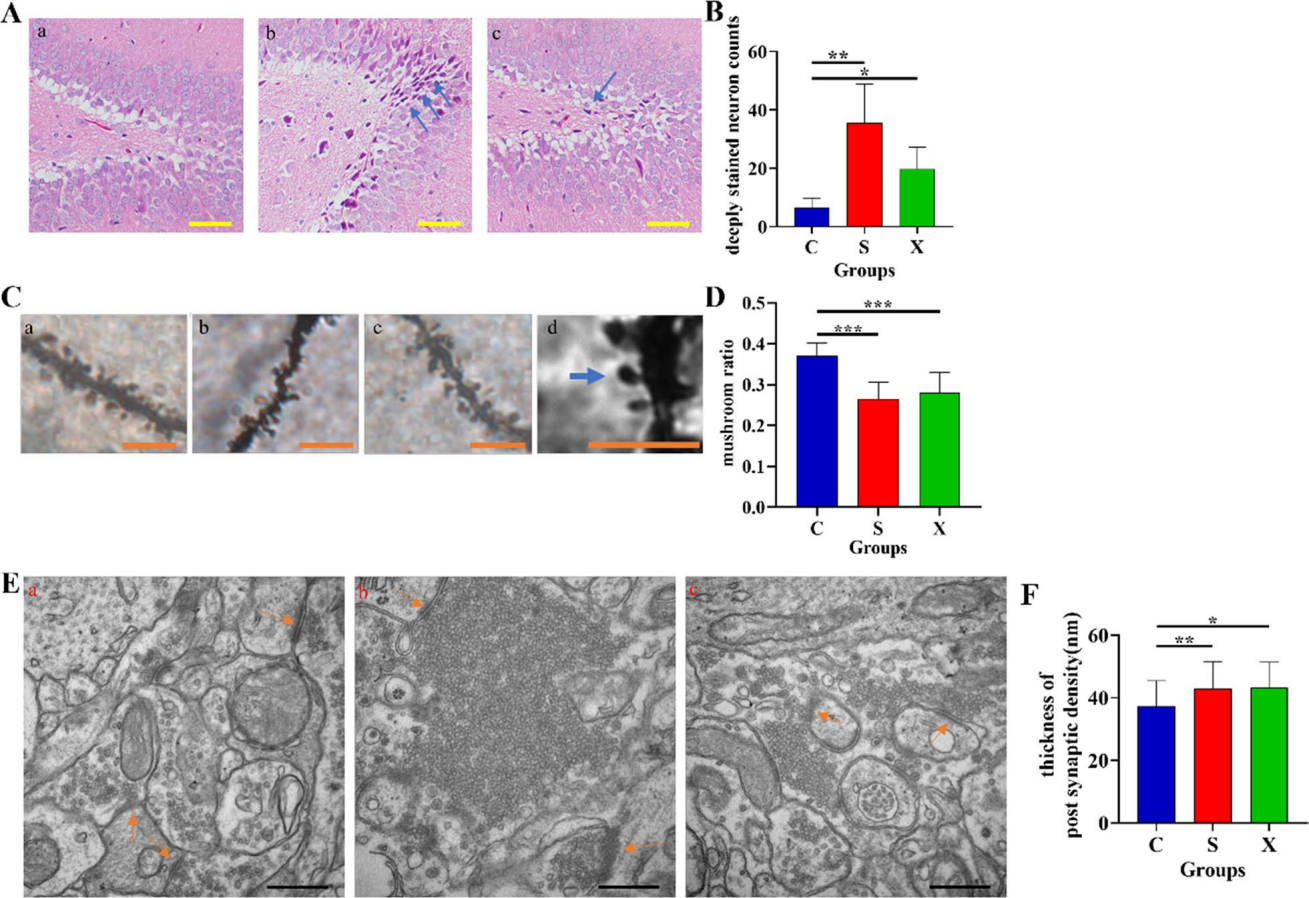
Changes in hippocampal structure after microwave exposure. **A** Microstructure of the DG region of the hippocampus of rats (**a** group C; **b** group S; **c** group X); **B** visible number of deeply stained cells in different groups; **C** dendritic spine staining of granule cells in the hippocampus of rats (**a** group C; **b** group S; **c** group X; **d** schematic diagram of mature dendritic spines); **D** proportion of mature dendritic spines in granule cells in the hippocampal region of rats in each group; **E** ultrastructure of the hippocampus of rats (groups C, S, X are shown in **a**–**c**); **F** thickness of PSD in different groups. * indicates *p* < 0.05, ** indicates *p* < 0.01; the blue arrows indicate damaged neurons, and the orange arrows indicate postsynaptic density. Scale bars = 1 mm for HE staining, 5 μm for Golgi staining and 500 nm for TEM.

In HE staining images, compared with the normal hippocampal organization in the C group (Fig. 3A-a), we found that neuron nucleus deep staining appeared in the DG area of the hippocampus after radiation in the S and X groups (Fig. 3A-b, c). Further quantitative analysis of the sections from this group showed that the hippocampal tissue of rats in both radiation groups exhibited structural damage (*p* = 0.001, *p* = 0.035, Fig. 3B).

Meanwhile, we examined the synaptic plasticity of granule cells in the hippocampal region of rats by Golgi staining (Fig. 3C) and found that the proportion of mature dendritic spines was significantly lower in the radiation groups than in the control group (Fig. 3D).

Moreover, we also examined the ultrastructure of neurons and synapses in the DG region. We found that vesicles accumulated at presynaptic terminals and that the postsynaptic density (PSD) was thickened in the radiated rats (Fig. 3E). Very numerous, abnormally packed vesicles in the presynaptic membranes were found in the exposure groups, especially for the S group (Fig. 3E-b, c). We also found that the PSD thickness of synapses increased significantly in the hippocampus of rats after two types of microwave exposure (*p* = 0.003, *p* = 0.023, Fig. 3 F).

### Long-term microwave radiation could lead to differential expression of proteins in the hippocampus

**Fig. 4:**
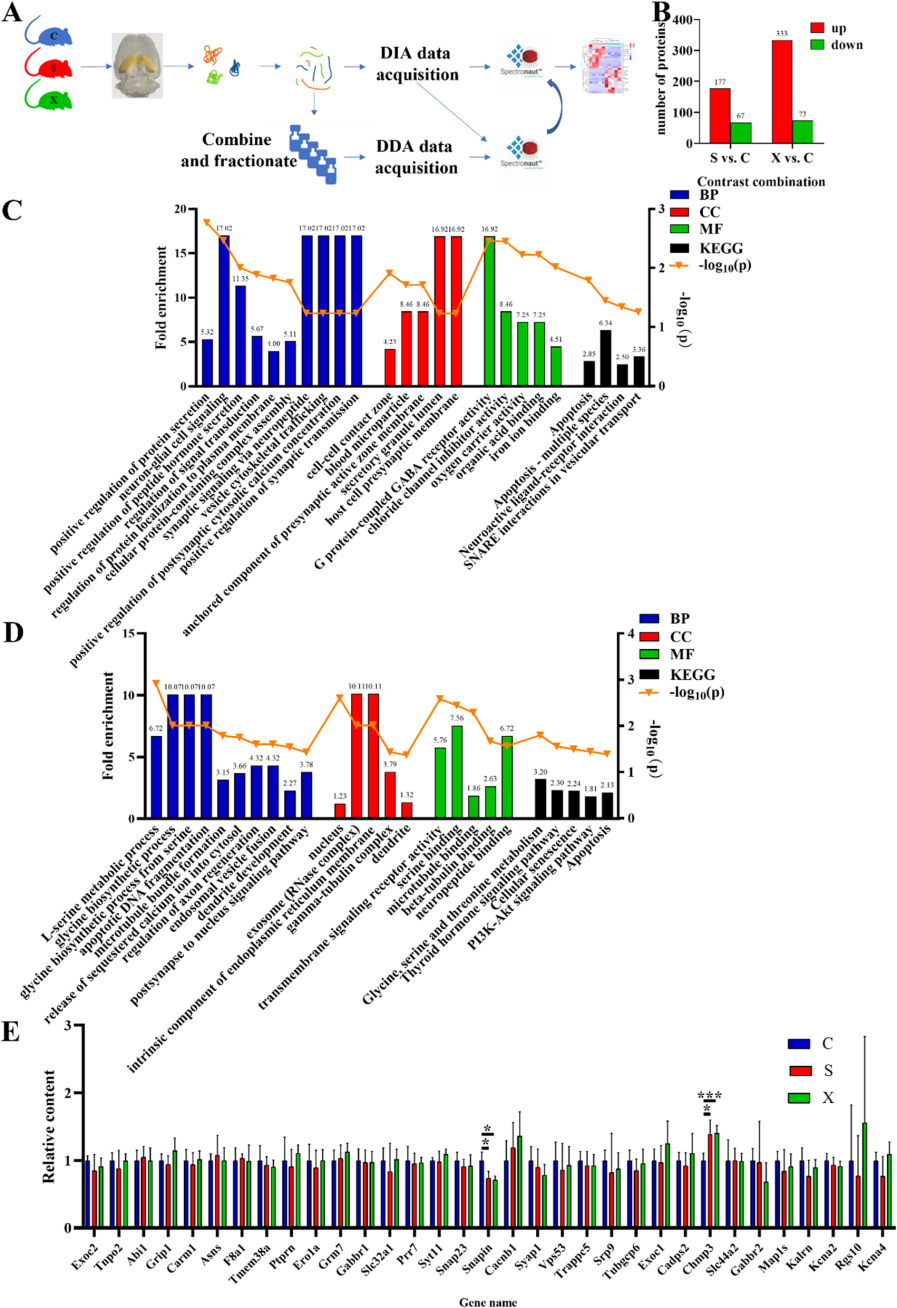
Proteomic analysis of the hippocampus after long-term microwave exposure. **A** Schematic diagram of the DIA proteomics method of hippocampal tissue. **B** Number of differentially expressed proteins. **C** GO and KEGG enrichment analysis results of differentially expressed proteins in hippocampal tissue between the C group and S group. **D** GO and KEGG enrichment analysis results of differentially expressed proteins in hippocampal tissue between the C group and X group. **E** Protein validation by PRM; * indicates *p* < 0.05, *** indicates *p* < 0.001

By DIA proteomic analysis of rat hippocampal tissue at 6 h after the last radiation (Fig. 4A), we found that there were 244 differentially expressed proteins between the S group and the C group, including 177 upregulated proteins and 67 downregulated proteins. There were 406 differentially expressed proteins between the X group and the C group, including 333 upregulated proteins and 73 downregulated proteins (Fig. 4B).

Bioinformatics analysis of differential proteins between the S and C groups was performed, and the enriched results of gene ontology (GO) analysis showed that the main biological process BP terms associated with differentially expressed proteins were *positive regulation of protein secretion, neuron-glial cell signaling, positive regulation of peptide hormone secretion, regulation of signal transduction, regulation of protein localization to plasma membrane, cellular protein-containing complex assembly, synaptic signaling via neuropeptide, vesicle cytoskeletal trafficking, positive regulation of postsynaptic cytosolic calcium concentration, positive regulation of synaptic transmission*, etc. The cellular component (CC) terms were *cell‒cell contact zone, blood microparticle, anchored component of presynaptic active zone membrane, secretory granule lumen, host cell presynaptic membrane*, etc. The molecular function (MF) terms included *G protein-coupled GABA receptor activity, lysophosphatidic acid phosphatase activity, chloride channel inhibitor activity, oxygen carrier activity*, and *organic acid binding* (Fig. 4C). KEGG pathways involving the differentially expressed proteins included *apoptosis, apoptosis—multiple species, neuroactive ligand‒receptor interaction*, and *SNARE interactions in vesicular transport* (Fig. 4C).

The analysis results of differentially expressed proteins between the X and C groups showed that the related BP terms included *L-serine metabolic process, glycine biosynthetic process, glycine biosynthetic process from serine, apoptotic DNA fragmentation, microtubule bundle formation, release of sequestered calcium ion into cytosol, regulation of axon regeneration, endosomal vesicle fusion, dendrite development*, and *postsynapse to nucleus signaling pathway*, among others. The CC terms related to the differentially expressed proteins included *nuclear body, nucleus, TRAPP complex, exosome (RNase complex), intrinsic component of endoplasmic reticulum membrane*, etc. The MF terms were focused on *transmembrane signaling receptor activity, serine binding, microtubule binding, beta-tubulin binding, neuropeptide binding*, etc. (Fig. 4D). The KEGG pathways involving the differentially expressed proteins were *glycine, serine and threonine metabolism*, *thyroid hormone signaling pathway*, *cellular senescence*, *PI3K-Akt signaling pathway*, *apoptosis*, etc. (Fig. 4D).

Then, we chose the differentially expressed proteins for the PRM test according to the relationship between protein function and microwave-related cognitive injuries. The BP and KEGG pathways were mainly considered to validate the candidate proteins. After validation by PRM, we found that SNARE-associated protein Snapin (gene name: Snapin) significantly decreased in the S and X groups (*p* = 0.016, *p* = 0.021), while the charged multivesicular body protein 3 (gene name: Chmp3) significantly increased in the S and X groups (*p* = 0.023, *p* < 0.001, Fig. 4E). The above results indicated that vesicle trafficking and synaptic vesicle recycling might be affected by long-term microwave exposure.

### Long-term microwave exposure could cause differential expression of serum exosome proteins

**Fig. 5:**
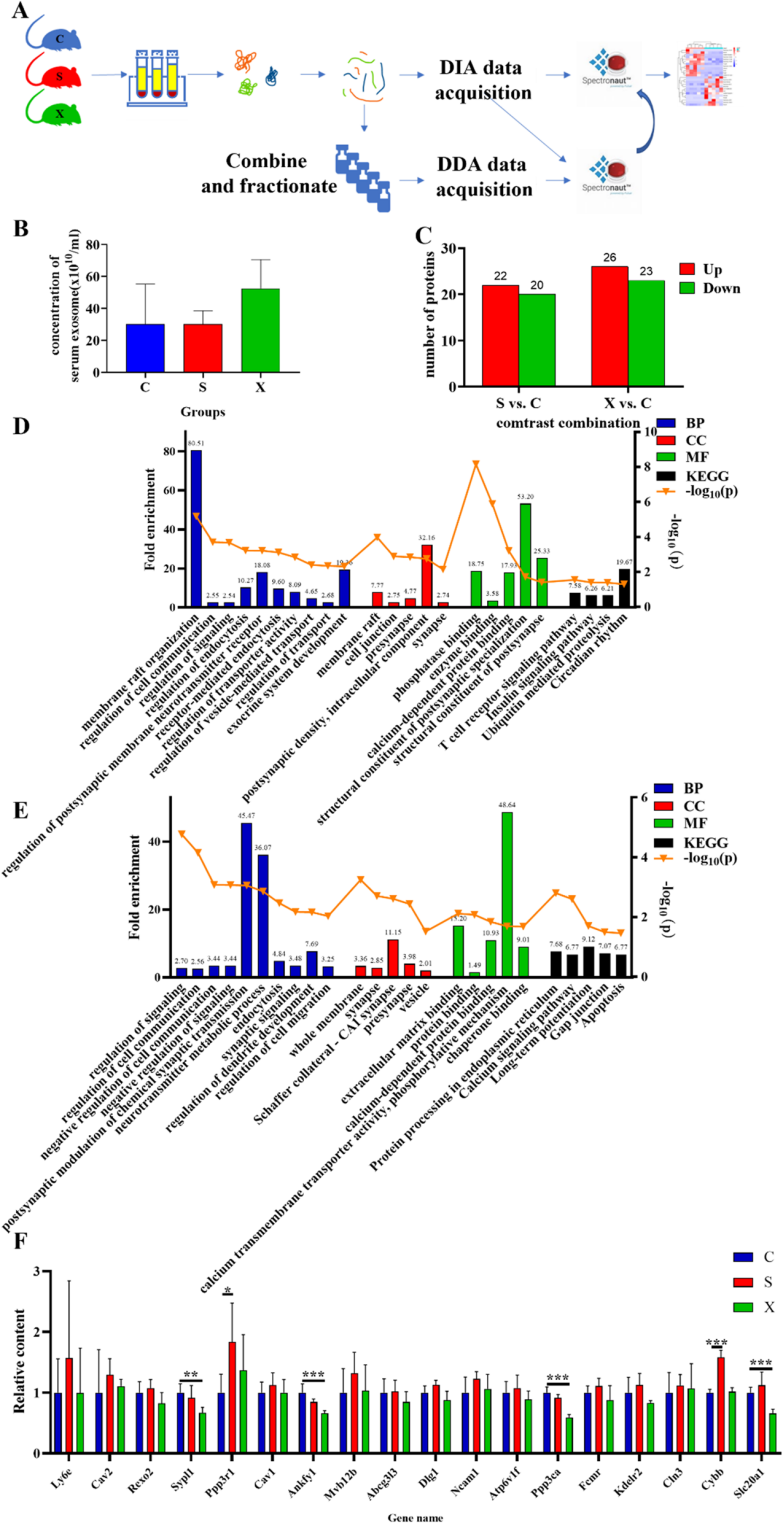
Proteomic analysis of serum exosomes after long-term microwave exposure. **A** Schematic diagram of the DIA proteomics method of serum exosomes. **B** Concentration of serum exosomes in each group. **C** Number of differentially expressed proteins. **D** GO and KEGG enrichment analysis results of differentially expressed proteins in serum exosomes between the C group and S group. **E** GO and KEGG enrichment analysis results of differentially expressed proteins in serum exosomes between the C group and X group. **F** Protein validation by MRM; * indicates *p* < 0.05, ** indicates *p* < 0.01, *** indicates *p* < 0.001

Exosomes were successfully extracted and identified by NTA, TEM and WB (Additional file 1: Fig. 2). According to the results of proteomic analysis and PRM validation of the hippocampus, we found that the process of vesicle trafficking might be affected after long-term microwave exposure. In addition, charged multivesicular body protein 3 was significantly increased. Because the multivesicular bodies (MVBs) are precursor components of exosomes, we also quantitatively analyzed the changes in proteins in exosomes from rat serum at 6 h after the last exposure (Fig. 5A). The concentration of exosomes showed an increasing trend in the X group, but there were no significant differences among the three groups (Fig. 5B).

Compared with the C group, the S group had 42 differentially expressed proteins, including 22 upregulated proteins and 20 downregulated proteins. Forty-nine differentially expressed proteins were found in the X group compared with the C group, including 26 upregulated proteins and 23 downregulated proteins (Fig. 5C). After the bioinformatics analysis, the BP of differential proteins in the S group included membrane raft organization, regulation of cell communication, regulation of signaling, regulation of endocytosis, regulation of postsynaptic membrane neurotransmitter receptor levels, receptor-mediated endocytosis, regulation of transporter activity, regulation of vesicle-mediated transport, regulation of transport, exocrine system development and so on. The CC terms associated with differentially expressed proteins in the S group were *membrane raft, cell junction, presynapse, postsynaptic density, intracellular component, synapse*, etc. The MF terms of the differentially expressed proteins in the S group were mainly focused on *phosphatase binding, enzyme binding, calcium-dependent protein binding, structural constituent of postsynaptic specialization, structural constituent of postsynapse*, etc. (Fig. 5D). The KEGG pathways of differentially expressed proteins for the S group were enriched in *T cell receptor signaling pathway, insulin signaling pathway, ubiquitin-mediated proteolysis, circadian rhythm*, etc. (Fig. 5D).

As for the differentially expressed proteins in the X group, the main BP terms were *regulation of signaling, regulation of cell communication, negative regulation of cell communication, negative regulation of signaling, postsynaptic modulation of chemical synaptic transmission, neurotransmitter metabolic process, endocytosis, synaptic signaling, regulation of dendrite development, regulation of cell migration*, etc. The CC terms were *whole membrane, synapse, Schaffer collateral-CA1 synapse, presynaptic vesicle*, etc. The MF terms included *extracellular matrix binding, protein binding, calcium-dependent protein binding, calcium transmembrane transporter activity, phosphorylative mechanism*, and *chaperone binding*, among others (Fig. 5E). Differential proteins involved in the KEGG pathway were enriched in protein processing in endoplasmic reticulum, calcium signaling pathway, long-term potentiation, gap junction, apoptosis and so on. (Fig. 5E).

After PRM detection, we found that calcineurin subunit B type 1 (gene name: Ppp3r1), cytochrome b-245 heavy chain (gene name: Cybb) significantly increased in the serum of rats in the S group (*p* = 0.029, *p* < 0.001), while synaptophysin-like 1 (gene name: Sypl1), ankyrin repeat and rabankyrin-5 (gene name: Ankfy1), protein phosphatase 3 catalytic subunit alpha (gene name: Ppp3ca) and sodium-dependent phosphate transporter 1 (gene name: slc20a1) significantly decreased in the X group (*p* = 0.002, *p* < 0.001, *p* < 0.001, *p* < 0.001, Fig. 5F).

## Discussion

A large number of studies have shown that microwave exposure could induce varying degrees of impairments of the central nervous system or functional injuries of other systems [26–29]. However, these studies mainly focused on the short-term effect of microwave radiation with a single band. 2.856 and 9.375 GHz microwaves are commonly used in occupational environments such as communications and meteorological monitoring. With an increasing amount of microwave equipment being used in our living environment, people would be affected by microwave exposure for a long period. Moreover, the differences caused by microwave radiation with different frequencies should also be given enough attention. Therefore, it was necessary to determine the biological effects after long-term microwave exposure at different frequencies. In addition, we attempted to identify biomarkers of exposure to different frequencies of microwave radiation.

The radiation duration in this study was based on the ICNIRP guidelines for Limiting Exposure to Electromagnetic Fields (2020 version), which established a standard time interval of 6 min for head health threats. Therefore, 6 min was used as a daily exposure time to explore the effects. Moreover, to determine whether the thermal effect played a role in the exposure period, a core temperature detection method was used in our study according to the previous literature [30]. The experimental results indicated that nonthermal effects were mainly involved in our study.

EEG was used in this study to reflect the physiological state of the cortex. We found a significant increase in shortwave power in the S group but only an increasing trend in the X group. Since slow waves tended to appear during low brain activation, the results implied that cortical activity in rats in the S group might be suppressed, which was similar to the findings of other researchers [30]. Moreover, the S-band microwaves induced significant changes in EEG signals, which might be related to the greater penetration depth of 2.856 GHz microwaves. In terms of cognitive tests, we examined spatial learning and memory abilities. The results of the MWM showed that the spatial learning and memory functions of the rats in the two exposure groups were impaired. Researchers also conducted behavior tests on spatial learning memory functions in animals after microwave exposure (2.45 GHz, 4 h/d, 45 d; 1 mW/cm^2^, 900 MHz, 3 h/d, 28 d; 10 mW/cm^2^, 1.5/4.3 GHz, once) [3, 16, 31] and found a significant reduction after microwave exposure. However, it has also been reported that spatial learning and memory functions were not affected in rats after 5.8 GHz microwave radiation for 4 h [32]. Our results were consistent with most studies, and the spatial learning and memory abilities significantly decreased in the two kinds of long-term microwave exposure. Further studies should be conducted in the future. In addition, we also excluded the possibility that differences in spatial learning and memory abilities were connected with athletic ability based on the detection of swimming speeds.

A number of studies have revealed the relationship between learning and memory abilities and the hippocampus [33, 34], including the role of different hippocampal subdivisions in spatial learning and memory function and its relationship to neurodegenerative diseases, such as Alzheimer’s disease. Moreover, many studies have confirmed that the hippocampus is one of the most important brain areas related to microwave radiation [8, 28, 35]. Based on the examination of the microstructure ultrastructure and synaptic structural plasticity and ultrastructure of the hippocampus, different degrees of damage were found in the hippocampus in the S and X groups. Neurons in the DG areas of the exposure groups showed similar changes with previous results about short-term microwave exposure [3], for example, the pyknotic nuclei in HE staining. In addition, dendritic spines are often used to assess neuroplasticity [32, 36]. In our study, we found that the proportion of mature dendritic spines in granule cells, the main cell type in the DG region, appeared significantly reduced in rats of both radiation groups. Interestingly, according to the TEM results, we found large abnormally packed vesicles in the presynaptic membranes, which indicated the abnormal synthesis and release process of vesicles after microwave exposure, especially for the S group. Therefore, we conducted proteomic detection of the hippocampus to determine the underlying mechanism.

Proteins are the executive molecules for various biological processes. As a new generation of proteomics that emerged with technological advances in mass spectrometry instrumentation, DIA-MS has better reproducibility and sensitivity than conventional DDA-MS [37]. Data from one study showed that the missing value of the DIA-MS (1.6%) was much lower than the missing value of the DDA-MS (55%) [38]. Therefore, we used the DIA method to identify sensitive molecules for microwave exposure. Surprisingly, our results found that the SNARE-associated protein Snapin significantly decreased after microwave exposure. Meanwhile, the charged multivesicular body protein 3 significantly increased.

The SNARE-associated protein Snapin is a component of the BLOC-1 complex. The BLOC-1 complex, in association with SNARE proteins, was also proposed to be involved in neurite extension, intracellular vesicle trafficking and synaptic vesicle recycling. It also modulates a step between vesicle priming, fusion and calcium-dependent neurotransmitter release through its ability to potentiate the interaction of synaptotagmin with SNAREs and the plasma membrane-associated protein SNAP25 [39]. Its phosphorylation state influences exocytotic protein interactions and may regulate synaptic vesicle exocytosis [40]. The charged multivesicular is the probable core component of the endosomal sorting required for transport complex III (ESCRT-III), which is involved in multivesicular body (MVB) formation and the sorting of endosomal cargo proteins into MVBs [41, 42]. According to the function of the two proteins, we believed that the 2.856 and 9.375 GHz microwaves decreased the process of vesicle trafficking and synaptic vesicle recycling and induced an increase in multivesicular bodies and synaptic vesicles, which was consistent with the changes observed by TEM. In addition, the BP and KEGG analyses of differentially expressed proteins in the hippocampus also indicated that vesicle cytoskeletal trafficking, SNARE interactions in vesicular transport and endosomal vesicle fusion were important processes taking place after microwave exposure. Multivesicular bodies are the precursors of exosomes. Therefore, according to the proteomics results of the hippocampus, we further studied the protein changes in serum exosomes.

Given that exosomes are capable of crossing the blood‒brain barrier and transferring information and exosomes in serum are more accessible than proteins in tissues, an increasing number of researchers have focused on the potential role of exosomes as biological markers [43–45]. Therefore, in this study, we first extracted exosomes from serum and tried to identify the differentially expressed proteins. According to the PRM verification results, we found differentially expressed proteins. The 2.856 GHz microwave long-term exposure could induce an increase in calcineurin subunit B type 1 and cytochrome b-245 heavy chain in serum exosomes. While the 9.375 GHz microwave long-term exposure induced a decrease in proteins (synaptophysin-like 1, ankyrin repeat and FYVE domain-containing 1, protein phosphatase 3 catalytic subunit alpha and sodium-dependent phosphate transporter 1) in serum exosomes. The results indicated that the different underlying proteins in serum exosomes were found after different frequencies. Although the behavior tests, EEG, and hippocampal structure showed similar changes after two different frequencies of microwave exposure, the protein changes in serum exosomes were totally different. After analyzing the protein functions, we concluded that the 2.856 GHz microwave exposure could increase the expression of two proteins, indicating that calcium sensitivity [46] and the transfer of the respiratory chain increased [47]. Excessive endocytosis and an increase in Ca^2+^ levels could induce calcium overload in cells and cause subsequent damage. While the 9.375 GHz microwave exposure decreased the expression of four proteins, indicating synaptophysin-like function, endocytosis, increased Ca^2+^ levels and decreased phosphate transport [48]. Although the same trend of the two markers after the two kinds of microwaves were found in the hippocampus, the proteins in the serum exosomes were totally different. The possible reasons were as follows. First, serum exosomes originate from various organs of the body. Second, the release processes of exosomes were different after different frequency microwaves. The possible mechanism might be related to the penetration depth and microwave energy.

Overall, we identified the differentially expressed proteins in the hippocampus and exosomes after microwave exposure, which might be able to serve as sensitive markers for microwave-induced injuries. Since exosomes in serum often come from various organs of the body, we need more tracing experiments in the future to determine the relationship of differentially expressed proteins between exosomes and the hippocampus. Knockout and overexpression animal models and additional assays, such as electrophysiological testing, are also needed to clarify the relationship between molecules and behavioral changes after microwave exposure.

## Conclusion

In summary, long-term microwave exposure (2.856 and 9.375 GHz, 6 min/d, 5 d/w, 6 w) led to different degrees of spatial learning and memory impairment, EEG disturbance, damage to hippocampal structure and differential expression of hippocampal tissue and serum exosomes. The SNARE-associated protein Snapin and charged multivesicular body protein 3 in the hippocampus could be used as sensitive markers of microwave exposure, and synaptic vesicle recycling was inhibited by long-term microwave exposure. Different proteins in serum exosomes were found after exposure to different frequency microwaves.

## Supplementary Information

**Additional file 1**. Supplementary files about DIA information, microwave equipment and identification of exosome.

## Data Availability

The data that support the findings of this study are available from the corresponding author, Ruiyun Peng, upon reasonable request.
