# Supplementary material for: Changes in cognitive function, synaptic structure and protein expression after long-term exposure to 2.856 and 9.375 GHz microwaves
Source: Cell Commun Signal. 2023 Feb 13;21:34. doi: 10.1186/s12964-022-01011-1 (PMC9926547; doi:10.1186/s12964-022-01011-1)
Supplement: Supplementary file 1 — Additional file 1. Supplementary files about DIA information, microwave equipment and identification of exosome. [file 12964_2022_1011_MOESM1_ESM.docx]

Supplement Table 1. DIA acquisition window

| **DIA window** | **Start m/z** | **End m/z** |
| --- | --- | --- |
| 1 | 350 | 405 |
| 2 | 405 | 429 |
| 3 | 429 | 453 |
| 4 | 453 | 466 |
| 5 | 466 | 479 |
| 6 | 479 | 492 |
| 7 | 492 | 505 |
| 8 | 505 | 518 |
| 9 | 518 | 531 |
| 10 | 531 | 544 |
| 11 | 544 | 557 |
| 12 | 557 | 570 |
| 13 | 570 | 583 |
| 14 | 583 | 596 |
| 15 | 596 | 609 |
| 16 | 609 | 622 |
| 17 | 622 | 635 |
| 18 | 635 | 648 |
| 19 | 648 | 661 |
| 20 | 661 | 674 |
| 21 | 674 | 687 |
| 22 | 687 | 700 |
| 23 | 700 | 713 |
| 24 | 713 | 726 |
| 25 | 726 | 739 |
| 26 | 739 | 752 |
| 27 | 752 | 765 |
| 28 | 765 | 778 |
| 29 | 778 | 796 |
| 30 | 796 | 814 |
| 31 | 814 | 832 |
| 32 | 832 | 855 |
| 33 | 855 | 878 |
| 34 | 878 | 901 |
| 35 | 901 | 924 |
| 36 | 924 | 947 |
| 37 | 947 | 981 |
| 38 | 981 | 1015 |
| 39 | 1015 | 1063 |
| 40 | 1063 | 1111 |
| 41 | 1111 | 1189 |
| 42 | 1189 | 1500 |


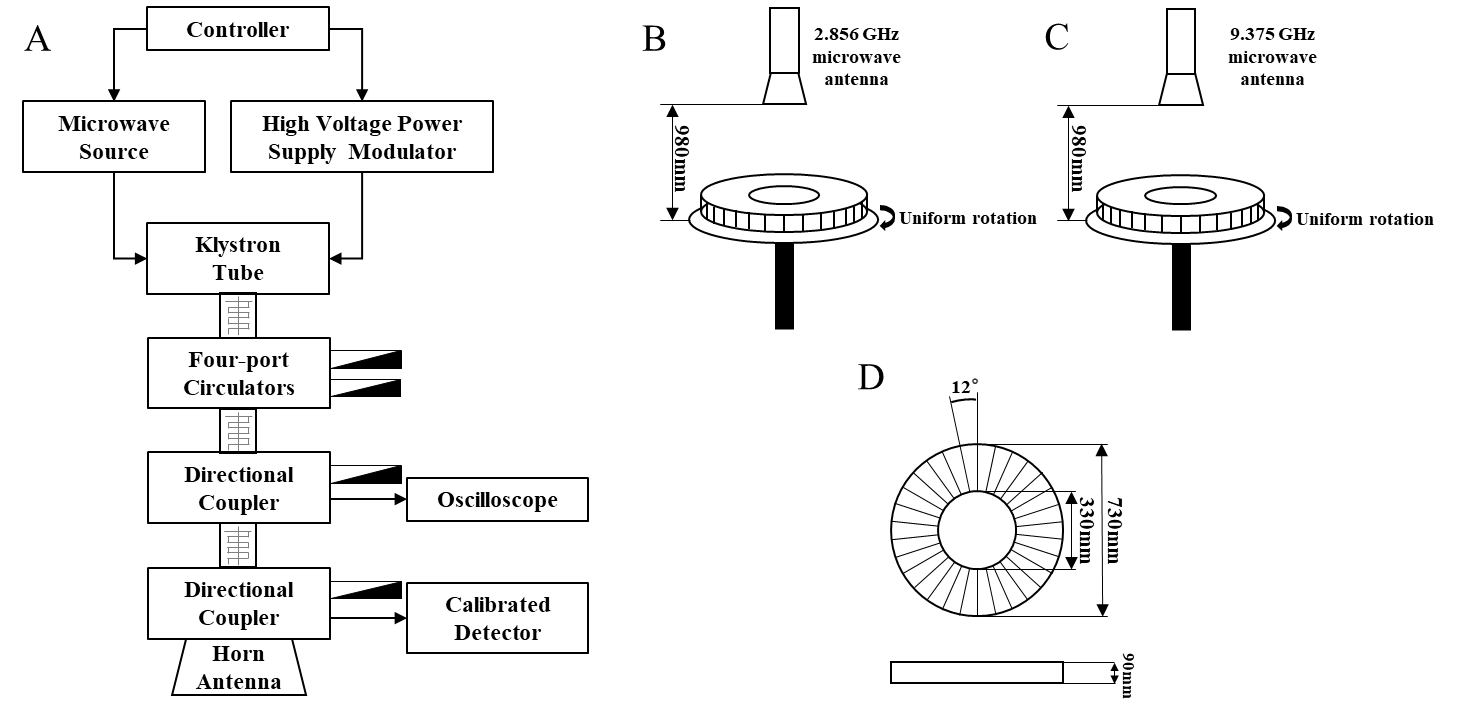


Supplemental Figure 1.

Schematic diagram of the microwave radiation experimental equipment. (A) Schematic diagram of the microwave radiation source. (B) Schematic diagram of the S-band microwave radiation process. (C) Schematic diagram of the X-band microwave radiation process. (D) Schematic diagram of the rat container. One rat was placed in each chamber, and the rats were subjected to whole-body exposure.

Question 3


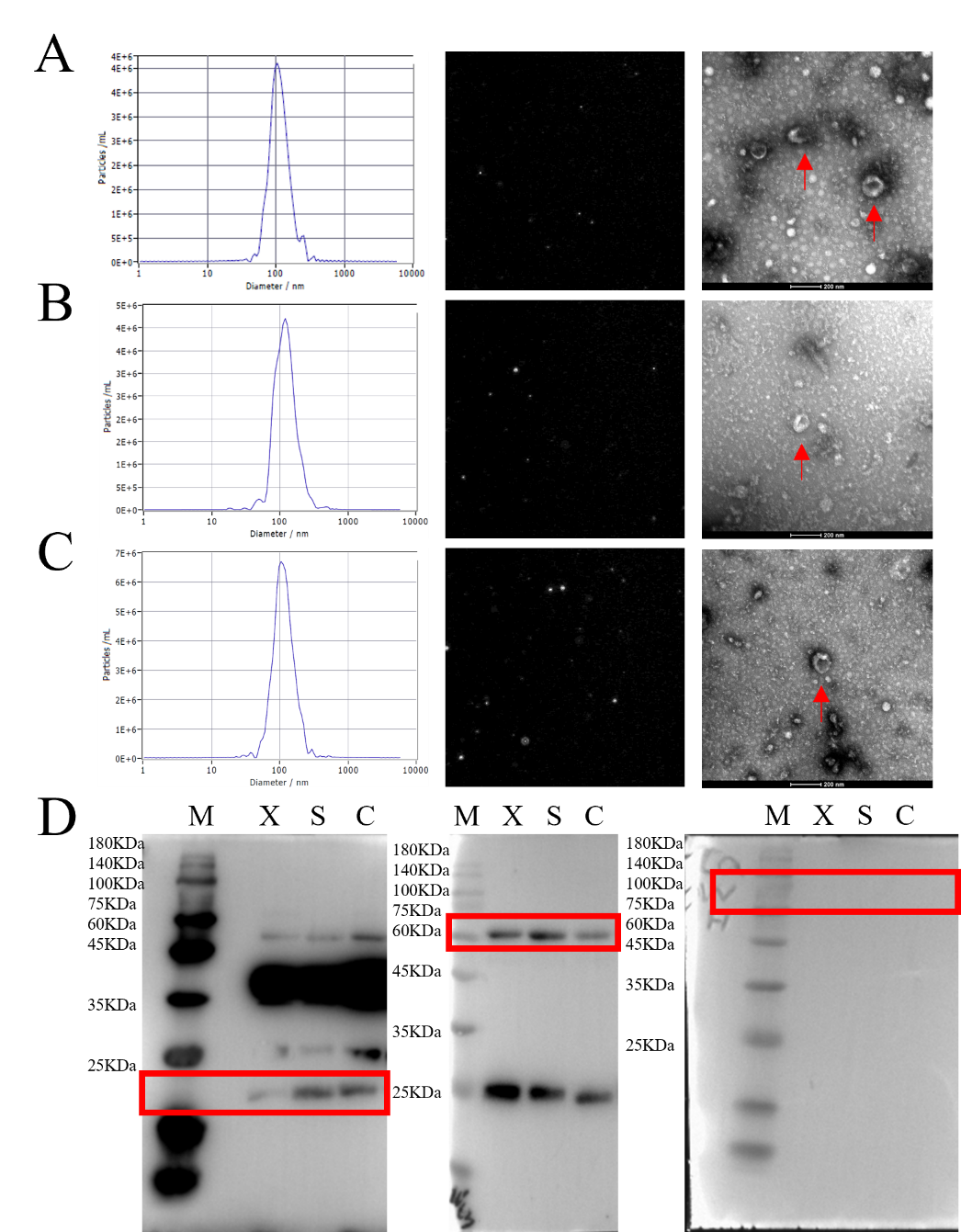


Supplemental Figure 2.

Acquisition of exosome information. (A-C) Serum exosome diameter distribution, nanoparticle tracking assay and TEM images of exosomes from rats in the C, S and X groups. (D) Western blots of CD9, CD63 and Calnexin.
